# Supplementary material for: EnzML: multi-label prediction of enzyme classes using InterPro signatures
Source: BMC Bioinformatics. 2012 Apr 25;13:61. doi: 10.1186/1471-2105-13-61 (PMC3483700; doi:10.1186/1471-2105-13-61)
Supplement: Addtional file 5 — The Java code to format the data files, evaluate and predict. The file enzml_java_code.tar.gz contains the Java code used to format database data to ARFF and XML formats, to execute cross and train-test (jackknife) evaluations and to record evaluation results to database. More information is included in the readme.txt file and the Javadoc files. The code can be used with a MySQL database. To use a different database software, other JDBC drivers might be required. [file 1471-2105-13-61-S5.gz › java_code/utils/doc/cern/colt/function/class-use/IntFunction.html]

Uses of Interface cern.colt.function.IntFunction


---


|  |  |  |
| --- | --- | --- |
| **Overview** \| **Package** \| **Class** \| **Use** \| **Tree** \| **Deprecated** \| **Index** \| **Help** | |  |
| PREV   NEXT | **FRAMES**    **NO FRAMES**     **All Classes** |  |


---


## **Uses of Interface cern.colt.function.IntFunction**

| Packages that use IntFunction | |
| --- | --- |
| **cern.jet.random** |  |
| **cern.jet.random.engine** |  |
| **uk.ac.ed.inf.utils.stats** |  |

Uses of IntFunction in cern.jet.random

| Classes in cern.jet.random that implement IntFunction | |
| --- | --- |
| `class` | `AbstractContinousDistribution`             Abstract base class for all continous distributions. |
| `class` | `AbstractDistribution`             Abstract base class for all random distributions. |
| `class` | `Pareto`             Pareto Distribution. |

Uses of IntFunction in cern.jet.random.engine

| Classes in cern.jet.random.engine that implement IntFunction | |
| --- | --- |
| `class` | `MersenneTwister`             MersenneTwister (MT19937) is one of the strongest uniform pseudo-random number generators known so far; at the same time it is quick. |
| `class` | `RandomEngine`             Abstract base class for uniform pseudo-random number generating engines. |

Uses of IntFunction in uk.ac.ed.inf.utils.stats

| Classes in uk.ac.ed.inf.utils.stats that implement IntFunction | |
| --- | --- |
| `class` | `PseudoTruncatedPareto`             A truncated pareto distribution which stores a big lists of power-law extracted random numbers. |
| `class` | `TruncatedPareto`             A utility class to extract from a truncated Pareto (power-law, long-tail) distribution. |

---


|  |  |  |
| --- | --- | --- |
| **Overview** \| **Package** \| **Class** \| **Use** \| **Tree** \| **Deprecated** \| **Index** \| **Help** | |  |
| PREV   NEXT | **FRAMES**    **NO FRAMES**     **All Classes** |  |


---
